# PTPRD/PTPRT mutation as a predictive biomarker of immune checkpoint inhibitors across multiple cancer types

**DOI:** 10.3389/fimmu.2022.991091

**Published:** 2022-09-29

**Authors:** Xiaoling Shang, Wengang Zhang, Xun Zhang, Miao Yu, Jingwen Liu, Yufeng Cheng, Bo Cheng

**Affiliations:** ^1^ Qilu Hospital of Shandong University, Cheeloo College of Medicine, Shandong University, Jinan, China; ^2^ Department of Medical Oncology, Qilu Hospital of Shandong University, Cheeloo College of Medicine, Shandong University, Jinan, China; ^3^ Shandong Provincial Third Hospital, Cheeloo College of Medicine, Shandong University, Jinan, China; ^4^ The Internet of Things, Shandong University of Science and Technology, Qingdao, China

**Keywords:** PTPRD, PTPRT, immune checkpoint inhibitors, pan cancer, tumor immune microenvironment

## Abstract

**Background:**

Immune checkpoint inhibitors (ICIs) are dramatically changing the treatment landscape of a variety of cancers. Nevertheless, the variability in ICI responses highlight the importance in identifying predictive biomarkers. PTPRD and PTPRT (PTPRD/PTPRT) are the phosphatases of JAK-STAT signaling, a critical pathway in anti-cancer immunity regulation. However, the pan-cancer association between PTPRD/PTPRT mutation and the efficacy of ICIs remains unclear across pan-cancer patients.

**Methods:**

We analyzed the association between PTPRD/PTPRT mutations and patient outcomes using clinical data and genomic mutations from TCGA pan-cancer cohort. Furthermore, the ICI-treatment cohort was used to evaluate the relationship between PTPRD/PTPRT mutation and the efficacy of ICIs. Another ICIs-treatment cohort was used to validate the findings. The TCGA pan-cancer dataset was analyzed to explore the correlation between PTPRD/PTPRT mutations and immune signatures. Moreover, we combined four factors to construct a nomogram model that could be used to predict the survival of pan-cancer patients receiving ICI treatment. The calibration curves and area under the curve were applied to assess the performance of the model.

**Results:**

PTPRD/PTPRT mutations were shown to be associated with a worse prognosis in TCGA cohort (*P* < 0.05). In the Samstein cohort, prolonged overall survival (OS) was observed in PTPRD/PTPRT mutant cancers, compared with wild-type cancers (mOS: 40.00 vs 16.00 months, HR = 0.570, 95%CI: 0.479-0.679, *P* < 0.0001). In the validation cohort, significant OS advantage was observed in PTPRD/PTPRT mutant patients (mOS: 31.32 vs 15.53 months, HR = 0.658, 95%CI: 0.464-0.934, *P* = 0.0292). Furthermore, PTPRD/PTPRT mutations were associated with a higher tumor mutational burden, MSI score, and TCR score (*P* < 0.0001). Enhanced immune signatures were found in the PTPRD/PTPRT mutant cancers (*P* < 0.05). Finally, we successfully established a nomogram model that could be used to predict the survival of NSCLC patients who received ICI treatment. Based on the risk score of the model, patients in the low-risk group showed a better mOS than those in the high-risk group (mOS: 2.75 vs 1.08 years, HR = 0.567, 95%CI: 0.492-0.654; *P* < 0.001).

**Conclusions:**

PTPRD/PTPRT mutations may be a potential biomarker for predicting ICI treatment responsiveness in multiple cancer types.

## Introduction

The development of immune checkpoint inhibitors (ICIs) has been a major milestone in the history of cancer therapy. Multiple ICIs, including antibodies that target programmed cell death protein 1 (PD-1), programmed death-ligand 1 (PD-L1), or cytotoxic T lymphocyte antigen 4 (CTLA4), have been approved for the treatment of a variety of advanced malignancies, including non-small cell lung cancer (NSCLC) (1), skin cutaneous melanoma (SKCM) (2), gastric cancer (3), and bladder urothelial carcinoma (BLCA) (4). ICIs have shown to be of significant clinical benefit. However, only a limited proportion of patients respond to ICIs as demonstrated by clinical trials and real-world evidence (5). Therefore, the variability in responses to ICIs highlight the importance of identifying predictive biomarkers.

Several biomarkers, including PD-L1 expression in tumor cells and immune cells (6), tumor mutational burden (TMB) (7), and microsatellite instability (MSI) (8), have been extensively investigated and demonstrated to be able to predict the response and the prognosis of patients treated with ICIs. However, these markers are still insufficient to identify patients who could benefit from ICI treatment. The positive or high expression of PD-L1 is the most widely used biomarker in clinical practice, and is associated with an improved response and survival when treated using ICIs. However, there are significant instances of when non-responders exert strong PD-L1 expression (9–11). The negative predictive value of PD-L1 is relatively low and about 20% of patients with negative PD-L1 expression respond to ICI treatment (12). Furthermore, PD-L1 does not serve as a pan-cancer biomarker. Its predictive value has been validated in several cancers, most notably NSCLC. Nevertheless, it is not reliable in predicting ICIs in RCC, SKCM, and other cancers (13). TMB is another biomarker that has received considerable attention and has been shown to be associated with ICI efficacy and may be a useful predictive biomarker. However, TMB expression is not always correlated with responsiveness of ICIs, which is a major challenge in TMB application (14). Therefore, more predictive biomarkers with a higher degree of sensitivity and stronger specificity are urgently needed to optimize ICIs treatment strategies.

Two members of the protein tyrosine phosphatase receptor (PTPR) family, PTPRT and PTPRD, have displayed significant mutation frequency in multiple malignancies. According to Du et al., PTPRT and PTPRD (PTPRD/PTPRT) mutations result in a decrease in phosphorylation, implying that they are loss-of-function mutations (15). Consistently, PTPRD/PTPRT mutations are associated with tumor progression and a worse patient prognosis (16). Sun et al. recently demonstrated that PTPRD mutations are a prognostic biomarker of NSCLC treated using ICIs (17). However, the clinical pan-cancer significance of PTPRD/PTPRT mutations in treated with ICIs remains unknown.

In this study, we performed a comprehensive analysis to evaluate the association between PTPRD/PTPRT mutations and the efficacy of ICIs across multiple cancers. Furthermore, investigations were conducted on the relationship between PTPRD/PTPRT mutations and immune signatures.

## Methods

### TCGA data

Data from TCGA PanCancer Atlas studies on 33 types of cancers, which included the prevalence analysis of PTPRD/PTPRT mutations and survival analysis, were downloaded from cBioportal for Cancer Genomics (https://www.cbioportal.org) (18). Survival analysis data was used to evaluate the impact of PTPRD/PTPRT mutations on prognosis.

### Data analysis of patients treated with ICIs

To explore the relationship between PTPRD/PTPRT mutations and the prognosis of pan-cancer patients receiving ICI treatment, data on a pan-cancer cohort, which included data on a total of 1,661 patients treated with ICIs, was downloaded from cBioportal (Samstein et al.) (19). The exclusion criteria used were as followed: patients with an unmatched somatic status (n=17), unknown primary cancer (n=87), cancer types that included only one patient (n=1). After filtering, 1,556 patients, including patients with BLCA (n= 212), breast cancer (n=43), colorectal cancer (CRC) (n= 110), esophagogastric cancer (n=125), glioblastoma (GBM) (n=116), head and neck cancer (HNSC) (n=138), SKCM (n=315), NSCLC (n= 346), and RCC (n=151) were included in this study. Another ICI-treated cohort (n=287) (Miao et al. and Huguo et al.) was creating and included overall survival data and genomic data, and was used as the validation set for this analysis (20, 21). Among them, 7 patients were treated with other concurrent therapy in addition to ICI therapy, one anal cancer patient, one small cell lung cancer patient, and one soft tissue sarcoma patient were excluded. Finally, a total of 277 patients were enrolled in the validation set, including patients with BLCA (n=27), HNSC (n=10), SKCM (n= 186), and NSCLC (n=54).

### TMB, MSI and TCR score analysis

The pan-cancer data was used to analyze the relationship between PTPRD/PTPRT mutations and nonsynonymous mutation counts, MSI sensor score, MSI Microsatellite Analysis for Normal-Tumor InStability (MANTIS) score, which were also downloaded from the cBioportal. T-cell receptor (TCR) richness score and TCR shannon score were obtained from a previous study led by Thorsson et al. (22). Co-mutation analysis was performed using TCGA PanCancer Atlas studies in the cBioportal database.

### Immune-related signature analysis

To investigate the association between anti-tumor immunity and PTPRD/PTPRT mutations, we evaluated immune signatures, immune cell infiltration, and immunity-related genes in TCGA pan-cancer cohort. The pan-cancer RNA-seq FPKM data were downloaded from UCSC Xena data portal (https://xenabrowser.net). A total of 29 immune signatures were retrieved from previous studies (23). Single simple gene set enrichment analysis (ssGSEA) was conducted to quantify the enrichment levels of the 29 immune signatures in each sample using the “GSVA” R package (24). In addition, the infiltration levels of 22 immune cells were analyzed using the CIBERSORT web portal (https://cibersort.stanford.edu/) using normalized gene expression data (25). Immunostimulator related genes, immunoinhibitor related genes, MHC molecule related genes, chemokine related genes, and receptor related genes were obtained from Thorsson et al. (22). Moreover, a gene set enrichment analysis (GSEA) was performed to compare differences in the activities of Kyoto Encyclopedia of Genes and Genomes (KEGG) between PTPRD/PTPRT mutated genes and wild type (WT) (26).

### Construction of a nomogram model to predict the benefits of ICIs

**Figure 1:**
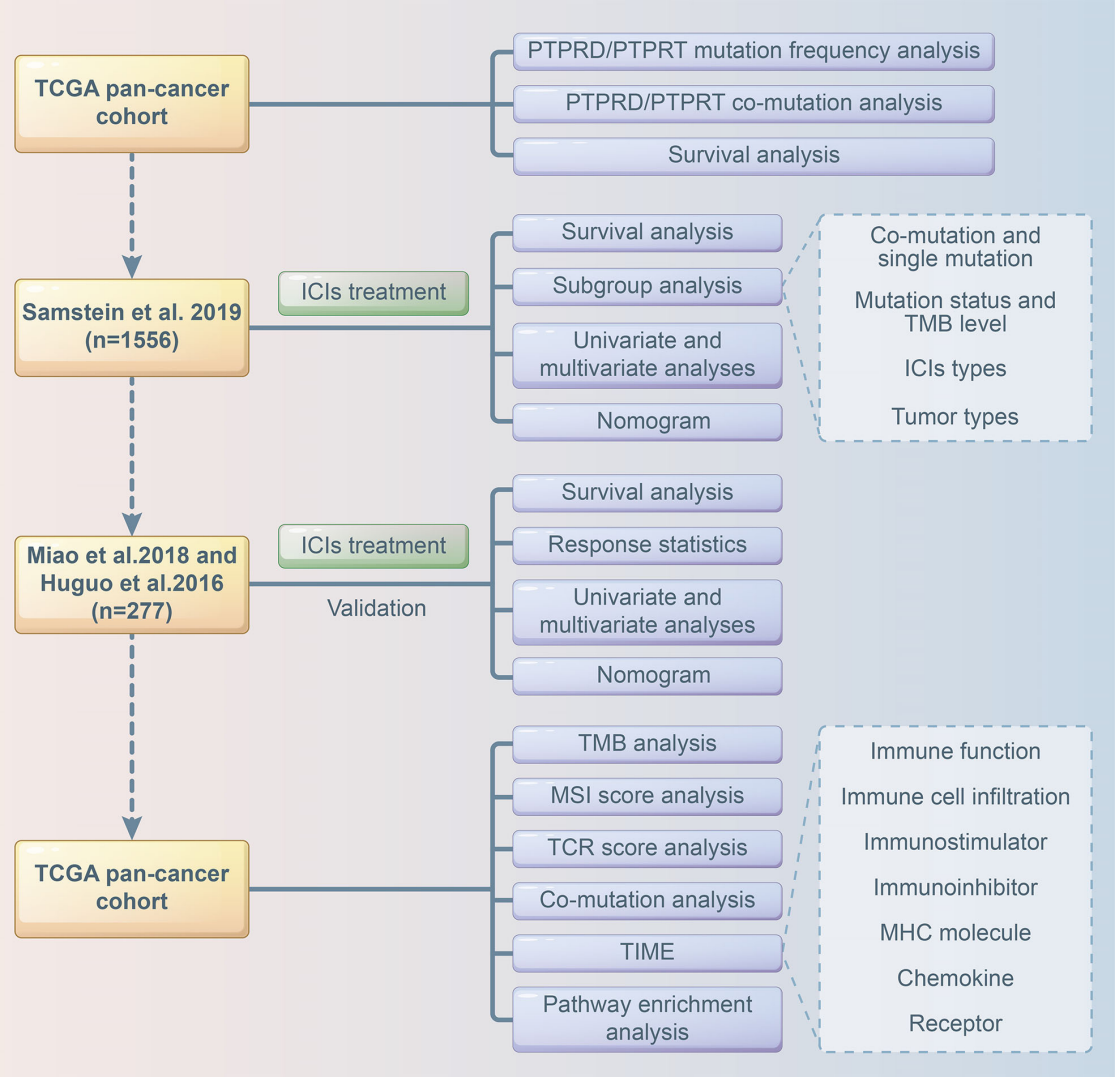
Flowchart of the study design.

Multivariate Cox analysis was performed to screen for factors that were significantly associated with the survival of patients receiving ICI treatment in the Samstein cohort. Cancer type, TMB, treatment type, and PTPRD/PTPRT mutations were included to construct a predictive model using the “rms”, “survival”, “survminer”, “timeROC” packages of R software. A calibration curve of the nomogram model was used for internal verification. The risk score was calculated based on the regression coefficient, and the patients were divided into a low-risk group and high-risk group based on the cutoff value of the risk score. The Miao et al. and Huguo et al. cohort was used as an external cohort to further validate the model. The flowchart of the study design is presented in **Figure 1**.

### Ethics statement

Ethical approval was not required since data included in the study were obtained from public databases.

### Statistical analysis

The Kaplan-Meier curve analysis of OS, progression-free survival (PFS), disease-specific survival (DSS), and disease-free survival (DFS) were performed using the log-rank test. The Chi-square test, Student’s t-test, and Fisher’s exact test were used to analyze the association between PTPRD/PTPRT mutations and various clinical characteristics of patients treated with ICIs. The univariate and multivariate Cox regression models were performed to analyze the effect of the variables, including age, sex, cancer type, treatment type, TMB and PTPRD/PTPRT mutation status, on clinical prognosis among pan-cancer patients treated with ICIs. Fisher’s exact test was used to assess the association between PTPRD/PTPRT mutation status and response to ICI treatment. Durable clinical benefit (DCB) was defined as complete response (CR), partial response (PR), or stable disease (SD) lasting longer than 6 months; no durable benefit (NDB) was defined as progression of disease (PD) or SD lasting less than 6 months. Spearman correlation coefficient was calculated to analyze the correlation between median TMB levels and PTPRD/PTPRT mutation frequency across multiple cancers. The Kruskal-Wallis test was performed to analyze the association between PTPRD/PTPRT mutation status and TCR score. A P-value less than 0.05 was considered to indicate statistical significance. All statistical analyses were performed using R software (version 4.1.2) and Graphpad prism software (version 8.0).

## Results

### PTPRD/PTPRT mutations in TCGA pan-cancer cohort

**Figure 2:**
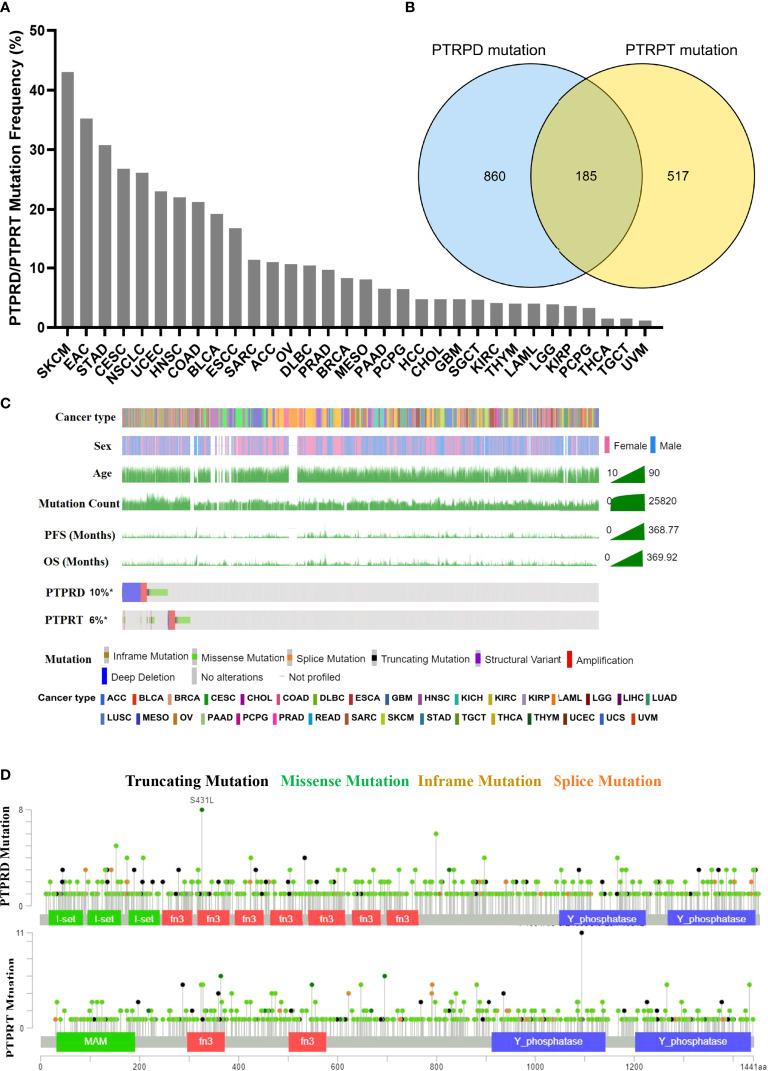
Mutational landscape of PTPRD/PTPRT in TCGA pan-cancer cohort. **(A)** Mutation frequency of PTPRD/PTPRT across tumors. **(B)** Co-mutation of PTPRD and PTPRT in pan-cancer. **(C)** The relationship between PTPRD/PTPRT mutations and clinical characteristics, including tumor type, gender, age, PFS and OS. **(D)** The subtypes and distribution of PTPRD and PTPRT mutations.

The pan-cancer frequency of PTPRD or PTPRT (PTPRT/PTPRD) mutations was analyzed using data from TCGA. The top five cancers with a high frequency of PTPRT/PTPRD mutations were SKCM (43.02%), esophagogastric adenocarcinoma (EAC) (35.21%), undifferentiated stomach adenocarcinoma (STAD) (30.77%), cervical squamous cell carcinoma (CESC) (26.77%), and NSCLC (26.12%), as shown in **Figure 2A**. Moreover, the results showed that a total of 185 patients harbored both PTPRD and PTPRT mutations (**Figure 2B**). The mutational landscape of PTPRD/PTPRT and its relationship with clinical characteristics are shown in **Figure 2C**. The lollipop plot depicted the pan-cancer distribution of different types of PTPRD/PTPRT mutations, with missense mutations being the most common subtype (**Figure 2D**).

### Association between PTPRD/PTPRT mutations and the survival of patients in TCGA pan-cancer cohort

**Figure 3:**
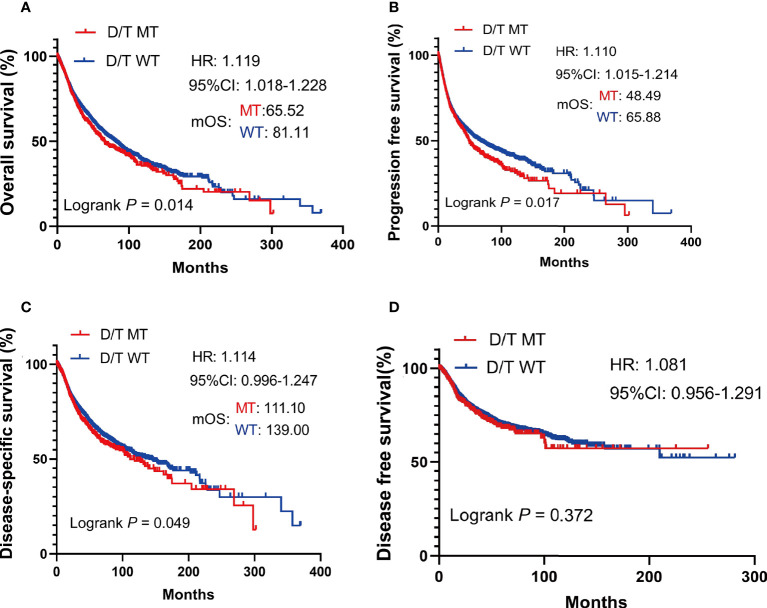
The survival significance of PTPRD/PTPRT mutation in TCGA pan-cancer cohort. Overall survival (OS) **(A)**, progression free survival (PFS) **(B)**, disease specific survival (DSS) **(C)**, and disease-free survival (DFS) **(D)** analysis were performed using the TCGA pan-cancer cohort according to PTPRT/PTRTD mutation status.

In TCGA pan-cancer cohort, survival analysis showed that the OS was worse in patients with PTPRD/PTPRT mutations than in the WT (mOS, 65.52 vs 81.11 months, HR = 1.119, 95%CI: 1.018-1.228; *P* = 0.014) (**Figure 3A**). The PFS (mPFS: 48.49 vs 65.88 months, HR = 1.110, 95%CI: 1.015-1.214; *P* = 0.017) (**Figure 3B**) and DSS (mDSS: 111.10 vs 139.00 months, HR =1.114, 95%CI: 0.996-1.247; *P* = 0.049) (**Figure 3C**) were also significantly worse in patients with PTPRD/PTPRT mutations. However, no significant difference was observed in disease free survival (DFS) between the mutation and WT groups (HR = 1.081. 95%CI: 0.956-1.291; *P* = 0.372) (**Figure 3D**). These findings indicate that patients with PTPRD/PTPRT mutations without ICIs treatment may be a pan-cancer risk factor.

### Association between PTPRD/PTPRT mutations and the survival of patients treated with ICIs in the Samstein cohort

To investigate the impact of PTPRD/PTPRT mutations on the efficacy of ICIs, genomic and clinical data on samples obtained from 1,556 pan-cancer patients treated with ICIs, including anti-PD-(L)1 monotherapy, anti-CTLA-4 monotherapy and anti-PD-(L)1 in combination with anti-CTLA-4 therapy, were retrieved from the cBioportal. As shown in **Table 1**, patients with an age ≥ 65 years (*P* = 0.044) and SKCM (*P* < 0.001) showed higher frequencies of PTPRD/PTPRT mutations. Meanwhile, patients with PTPRD/PTPRT mutations had a significantly higher proportion of high-TMB, compared with WT patients (*P* < 0.001, 90.4% vs 43.6%).

**Table 1:** Relationship between PTPRD/PTPRT mutation and clinicopathological characteristics in ICI-treared cohort (Samstein et al.).

Variables	D/T mutation (n=249)	Non-D/T mutation(n=1307)	*P*
**Age**			0.044
< 65	123 (49.4)	739 (56.5)	
≥65	126 (50.6)	568 (43.5)	
**Sex**			0.369
Female	32 (12.9)	191 (14.6)	
Male	167 (67.1)	816(62.4)	
**Cancer type**			<0.001
NSCLC	67 (26.9)	279 (21.3)	
SKCM	108 (43.4)	207 (15.8)	
Others	74 (29.7)	821(62.8)	
**Treatment**			<0.001
Monotherapy	204 (81.9)	197 (15.1)	
Combination	45 (18.1)	1110 (84.9)	
**TMB**			<0.001
Low	24 (6.6)	737 (56.4)	
High	225 (90.4)	570 (43.6)	
**Median OS (months)**	15.00	10.00	

**Figure 4:**
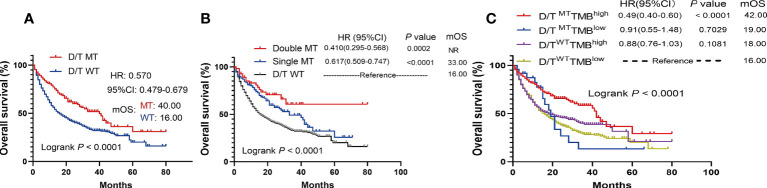
The survival significance of PTPRD/PTPRT mutation in ICIs-treated cohort (Samstein et al., n = 1556). **(A)** Association between PTPTD/PTRT mutation and OS in pan-cancer treated with ICIs. **(B)** Effect of PTPRD/PTPRT single mutation versus co-mutation on patients treated with ICIs. **(C)** Survival analysis was performed based on PTPRD/PTPRT mutation status and TMB level.

The survival analysis showed that patients with PTPRD/PTPRT mutations had a significantly longer OS than WT patients (mOS: 40.00 vs 16.00 months, HR = 0.570, 95%CI: 0.479-0.679; *P* < 0.0001) (**Figure 4A**). Moreover, the survival analysis of co-mutations and single mutation of the PTPRD/PTPRT gene was performed. The results showed that patients with PTPRD and PTPRT double mutations (mOS: not reached (NR) vs 16.00 months, HR = 0.410, 95%CI: 0.295-0.568; *P* = 0.0002) or single mutations (mOS: 33.00 vs 16.00 months, HR = 0.617, 95%CI: 0.509-0.747; *P* < 0.0001) experienced a longer survival duration, compared with WT patients (**Figure 4B**). Additionally, patients were classified as D/T^Mut^TMB^high^, D/T^Mut^TMB^low^, D/T^WT^TMB^high^, and D/T^WT^TMB^low^ based on their PTPRD/PTPRT mutation status and TMB level. As expected, D/T^Mut^TMB^high^ patients showed the longest OS among all groups (**Figure 4C**). In patients with a high-TMB level, PTPRD/PTPRT mutations could be used to successfully identify patients with a survival advantage (HR = 0.49; 95%CI: 0.40-0.60, *P* < 0.0001, mOS: 42.00 months), compared with WT patients (**Figure 4C**). However, patients with a low-TMB level did not show a statistically significant difference in survival based on PTPRD/PTPRT mutations (HR = 0.91; 95%CI: 0.55-1.48, *P* = 0.7029, mOS: 19.00 months) and PTPRD/PTPRT WT status (mOS: 16.00 months) (**Figure 4C**).

### Subgroup analysis of patients treated with ICIs in the Samstein cohort

**Figure 5:**
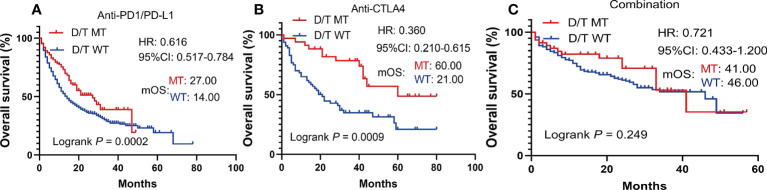
Subgroup survival analysis based on the ICIs types. **(A)** Survival analysis in patients treated with anti-PD-(L)1. **(B)** Survival analysis in patients treated with anti-CTLA4. **(C)** Survival analysis in patients treated with anti-PD-(L)1 in combination with anti-CTLA4.

To further investigate whether the prediction ability of PTPRD/PTPRT mutations was affected by the type and treatment modalities of the ICIs, a subgroup survival analysis was performed. In patients with PTPRD/PTPRT mutations, better OS was still observed in the anti-PD-(L)1 treatment group (mOS, mutation vs WT: 27.00 vs 14.00 months, HR =0.616, 95%CI: 0.517-0.784; *P* = 0.0002) (**Figure 5A**) and the anti-CTLA4 group (mOS, mutation vs WT: 60.00 vs 21.00 months, HR =0.360, 95%CI: 0.210-0.615; *P* = 0.0009) (**Figure 5B**). However, no OS advantage was found in PTPRD/PTPRT mutant patients receiving anti-PD-(L)1 and anti-CTLA4 combination treatment (mOS, mutation vs WT: 41.00 vs 46.00 months, HR = 0.721, 95%I: 0.433-1.200; *P* = 0.2492) (**Figure 5C**).

We further analyzed the effects of different mutation subtypes on the efficacy of ICI treatment. The results showed that the OS of patients with PTPRD/PTPRT mutations, especially with missense mutations, was significantly better than that of WT patients following ICI treatment (both *P* < 0.001) (**Supplementary Figure 1**). To determine the influence of PTPRD/PTPRT mutations on different types of cancers, a subgroup analysis was performed based on tumor type. The results showed that the PTPRD/PTPRT mutant patients experienced significantly longer OS than WT patients with NSCLC (*P* = 0.0292) (**Supplementary Figure 2A**) and SKCM (*P* = 0.0018) (**Supplementary Figure 2B**). A trend towards an OS benefit was observed in PTPRD/PTPRT mutant patients with the BLCA (**Supplementary Figure 2C**), CRC (**Supplementary Figure 2D** **)**, EAC (**Supplementary Figure 2E**), and HNSC (**Supplementary Figure 2F**), despite a statistically significant difference not being found. No significant survival was observed in GBM (**Supplementary Figure 2J**) or RCC (**Supplementary Figure 2H**) (both *P* > 0.05).

### Validation of the effect of PTPRD/PTPRT mutations on the benefit of ICIs in the Miao et al. and Huguo et al. cohorts

**Figure 6:**
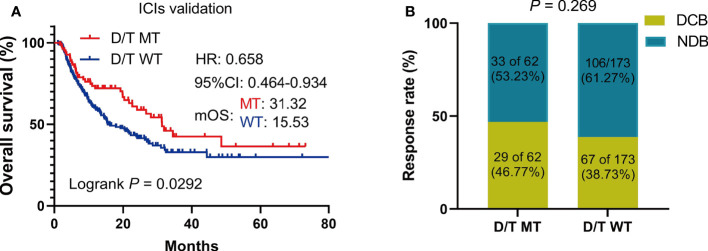
The survival significance of PTPRD/PTPRT mutation in another ICIs-treated cohorts (Miao et al. and Huguo et al., n = 277). **(A)** Association between PTPTD/PTRT mutation and OS in pan-cancer treated with ICIs. **(B)** Response to ICIs in PTPRD/PTPRT mutated versus WT patients. DCB: durable clinical benefit; NDB: no clinical benefit.

To further validate the predictive value of PTPRD/PTPRT mutations in terms of ICI efficacy, clinical and genetic mutation data was collected from another ICIs-treated cohort used by Miao et al. and Huguo et al. The clinical characteristics of patients included in this cohort is summarized in **Supplementary Table 1**. Similar to the above findings, patients with PTPRD/PTPRT mutations had significantly longer OS, compared with WT patients (mOS: 31.32 vs 15.53 months; HR = 0.658, 95% CI: 0.4637-0.9338, *P* = 0.029) (**Figure 6A**). In addition, patients who harbor PTPRD/PTPRT mutations had a higher DCB ratio than WT patients (46.77% vs 38.73%) (**Figure 6B**). On the other hand, the NDB ratio of the WT group was higher (53.23% vs 61.27%) (**Figure 6B**).

### Independent predictive value of PTPRD/PTPRT mutations for the survival of pan-cancer patients receiving ICIs

To determine the independent predictive factors that can be used to predict ICI efficacy, univariate and multivariate COX regression analyses were performed. First, in the ICI-treatment cohort reported by Samstein et al., tumor type and treatment strategies could be used to independently predict the survival benefit of the ICIs **(** **Table 2** **)**. Most importantly, the univariate (HR = 0.585, 95% CI: 0.472-0.725, *P* < 0.0001) and multivariate (HR = 0.703, 95% CI: 0.557-0.887, *P* < 0.003) COX analyses showed that patients with PTPRD/PTPRT mutations had a better prognosis than WT patients, indicating that PTPRD/PTPRT mutations are an independent prognostic factor in pan-cancer patients treated with ICIs (**Table 2**). Further validation was performed using the validation cohort reported on by Miao et al. and Huguo et al. In line with the above mentioned results, PTPRD/PTPRT mutations were also determined to be an independent pan-cancer predictive factor for patients treated with ICIs (univariate: HR = 0.657, 95% CI: 0.449-0.961, *P* = 0.030; multivariate: HR = 0.612, 95% CI: 0.417-0.896, *P* = 0.012) (**Supplementary Table 2**).

**Table 2:** Univariate and multivariate analyze Cox regression in ICI-treated cohort (Samstein et al.).

Variable	Univariate analyses			Multivariate analyses		
	HR	95% CI	*P*	HR	95% CI	*P*
**Age**				NI		
< 65	0.983	0.854-1.131	0.808			
>= 65	Reference					
**Sex**						
Female	Reference			NI		
Male	0.902	0.781-1.042	0.162			
**Cancer type**						
NSCLC	Reference			Reference		
SKCM	1.268	1.078-1.492	0.004	1.330	1.128-1.567	0.001
Others	0.514	0.420-0.629	< 0.0001	0.629	0.509-0.777	< 0.0001
**Treatment**						
Monotherapy	Reference			Reference		
Combination	0.565	0.454-0.702	< 0.0001	0.654	0.523-0.817	< 0.0001
**TMB**						
Low	Reference			Reference		
High	0.767	0.666-0.883	< 0.0001	0.864	0.744-1.003	0.054
**D/T mutation**						
No	Reference			Reference		
Yes	0.585	0.472-0.725	< 0.0001	0.703	0.557-0.887	0.003

NI, not included.

### Association between PTPRD/PTPRT mutations and the TMB, MSI score, or TCR

**Figure 7:**
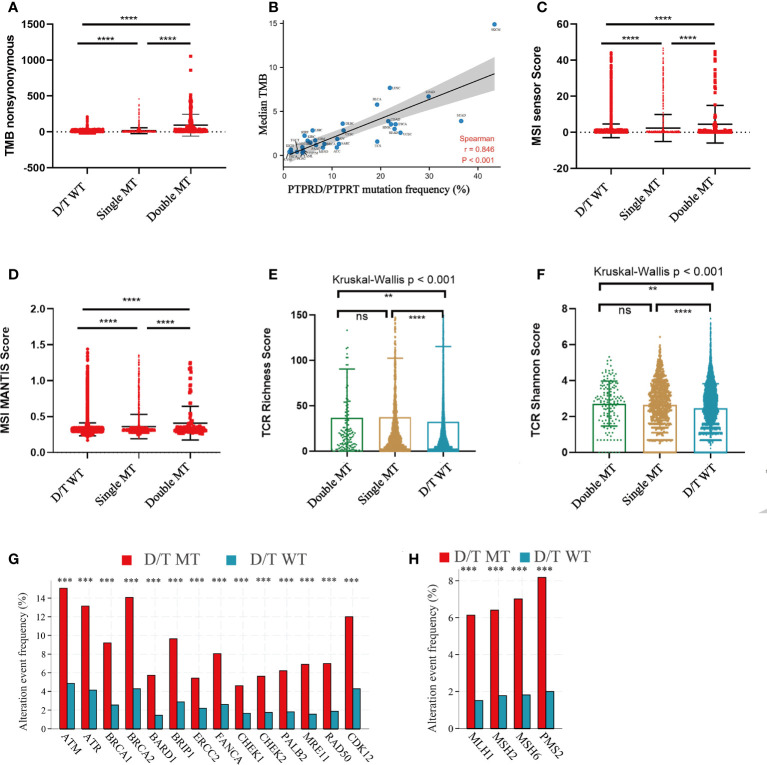
The correlation between PTPRD/PTPRT mutation and multiple predictive variables for ICIs. **(A)** TMB levels among PTPRD/PTPRT WT, single-mutant, and double-mutant tumors. **(B)** The correlation between PTPRD/PTPRT mutation frequency and median TMB score across tumors. **(C)** MSI sensor scores among PTPRD/PTPRT WT, single-mutant, and double-mutant tumors. **(D)** MSI MANTIS scores among PTPRD/PTPRT WT, single-mutant, and double-mutant tumors. **(E)** TCR richness scores among PTPRD/PTPRT WT, single-mutant, and double-mutant tumors. **(F)** TCR Shannon scores among PTPRD/PTPRT WT, single-mutant, and double-mutant tumors. **(G)** The correlation between DDR-genes mutation and PTPRD/PTPRT mutation. **(H)** The correlation between MMR-gene mutation and PTPRD/PTPRT mutation (**P < 0.01, ***P < 0.001, ****P < 0.0001, ns, no significance). TMB, tumor mutational burden; MSI, microsatellite instability; MANTIS, Microsatellite Analysis for Normal-Tumor InStability; TCR, T-cell receptor; DDR, DNA damage response; MMR, mismatch repair.

To elucidate the underlying mechanisms of PTPRD/PTPRT mutations that affect ICIs efficacy, the relationship between PTPRD/PTPRT mutations and multiple indicators associated with ICI response were analyzed. First, the relationship between PTPRD/PTPRT mutations and TMB was explored. The results showed that TMB was significantly higher in the PTPRD/PTPRT mutant patients than in WT patients (*P* < 0.0001) (**Figure 7A**). Meanwhile, TMB was higher in patients with PTPRD and PTPRT double mutations than patients with a single mutation (*P* < 0.0001) (**Figure 7A**). Importantly, the spearman correlation analysis revealed a positive correlation between the frequencies of PTPRD/PTPRT mutations and the median TMB level across multiple cancers (correlation coefficient, 0.846; *P* < 0.001) (**Figure 7B**). Second, the association between PTPRD/PTPRT mutations and MSI was explored. The PTPRD/PTPRT mutant cancer patients had a significantly higher MSI sensor score, compared with WT patients (*P* < 0.0001) (**Figure 7C**). In addition, the MSI sensor score was significantly higher in the PTPRD and PTPRT double-mutant cancers than in the single-mutant cancers (*P* < 0.0001) (**Figure 7C**). The relationship between PTPRD/PTPRT mutations and MSI MANTIS score, a new method for effectively and accurately assessing MSI status, was explored whether there was a stronger reliability in the correlation between MSI score and PTPRD/PTPRT mutations. In line with the results of MSI sensor score, a higher MSI MANTIS score was found in PTPRD/PTPRT single-mutant or double-mutant cancers, compared with WT patients (*P* < 0.0001) (**Figure 7D**). Third, we explored the effect of PTPRD/PTPRT mutations on TCR diversity, which was strongly associated with the anti-tumor response. The results demonstrated that the TCR Richness Score was notably higher in both the PTPRD/PTPRT single-mutant and double-mutant cancers, than in WT patients (*P* < 0.001), while there was no statistical difference between PTPRD/PTPRT single-mutant and double-mutant patients (*P* > 0.05) (**Figure 7E**). Similarly, a higher TCR Shannon Score was observed in the PTPRD/PTPRT single-mutant or double-mutant cancers, compared with WT patients (*P* < 0.01) (**Figure 7F**). Fourth, the association between the DNA damage response (DDR) pathway gene mutations and PTPRD/PTPRT mutations were explored. Notably, fourteen DDR gene mutations were significantly more prevalent in PTPRD/PTPRT mutant cancer patients than in WT patients (all *P* < 0.001) (**Figure 7G**). Fifth, the frequency of four DNA mismatch repair (MMR) gene mutations were higher in the PTPRD/PTPRT mutant cancer patients, compared with WT patients (all *P* < 0.001) (**Figure 7H**). The specific statistical data on the DDR and MMR related genes were summarized in **Supplementary Table 3**. Collectively, TMB, MSI score, TCR score, DDR-gene mutations, and MMR-gene mutations were found to be positively associated with PTPRD/PTPRT mutations.

### Correlation between PTPRD/PTPRT mutations and immune signatures

**Figure 8:**
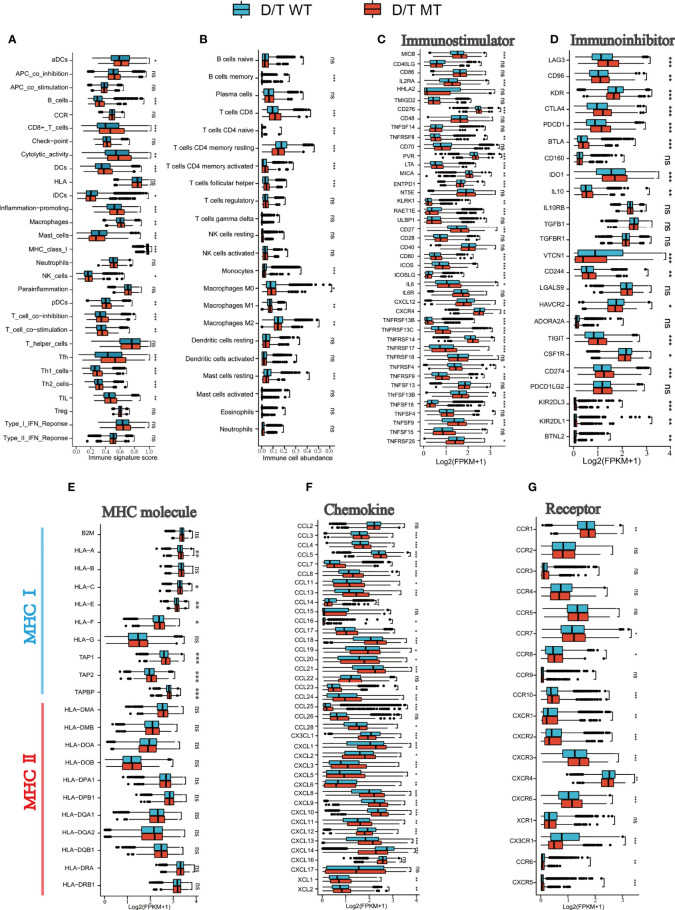
PTPRD/PTPRT mutation was associated with enhanced anti-tumor immunity in the TCGA cohort. **(A)** The correlation between PTPRD/PTPRT mutation and 29 immune signatures was analyzed using the ssGSEA method based on RNA-sequencing data. **(B)** Boxplot Profiling the infiltration of 22 immune cells between PTPRD/PTPRT mutant and WT tumors. **(C)** Analysis of the levels of immunostimulators between PTPRD/PTPRT mutant and WT tumors. **(D)** Analysis of the levels of immunoinhibitors between PTPRD/PTPRT mutant and WT tumors. **(E)** Analysis of the levels of MHC molecules between PTPRD/PTPRT mutant and WT tumors. **(F)** Analysis of the levels chemokines between PTPRD/PTPRT mutant and WT tumors. **(G)** Analysis of the levels receptors between PTPRD/PTPRT mutant and WT tumors (*P < 0.05, **P < 0.01, ***P < 0.001, ns, no significance).

To further elucidate the immune environment of the PTPRD/PTPRT mutant patients, the relationship between PTPRD/PTPRT mutations and immune signatures were analyzed. First, the enrichment levels of 29 immune signatures were evaluated between PTPRD/PTPRT mutant and WT cancers. The immune signature scores, such as CD8^+^ T cells, Tfh, B cells, DCs, cytotoxic activity, and pro-inflammation, were higher in PTPRD/PTPRT mutant patients than in WT patients (all *P* < 0.05) (**Figure 8A**). For further validation, the infiltration levels of 22 immune cells were analyzed. Most immune cells, including memory B cells, CD8^+^ T cells, Tfh, and macrophage M1 cells, were more abundant in PTPRD/PTPRT mutant cancer patients (all *P* < 0.05) (**Figure 8B**). In contrast, macrophage M2 cells were more abundant in WT cancer patients (*P* < 0.05) (**Figure 8B**). These findings indicate that an enhanced anti-tumor microenvironment was created by the PTPRD/PTPRT mutations. Second, most immuno-stimulators, such as MICB, MICA, CD80, ICOS, CD27, and various TNFSFs (all *P* < 0.05), were expressed at higher levels in PTPRD/PTPRT mutant cancers, compared with the WT (**Figure 8C**). In addition, several immune inhibitory molecules, including LAG3, CD96, CTLA4, PDCD1, BTLA, IDO1, CD274, and TIGIT, were enriched in PTPRD/PTPRT mutant cancers, all of which are crucial immune checkpoints for anti-tumor drugs (all *P* < 0.05) (**Figure 8D**). Third, major histocompatibility complex (MHC) performs crucial functions during the process of antigen presentation, and its absence is a major mechanism that contributing to tumor immune escape. As shown in **Figure 8E**, most MHC I molecules, including HLA-A, HLA-C, HLA-E, HLA-F, TAP1, TAP2, and TAPBP (all *P* < 0.05), were upregulated in PTPRD/PTPRT mutant patients, indicating enhanced anti-tumor immunity in PTPRD/PTPRT mutant patients. However, no significant differences were observed in levels of MHC II molecules (all *P* > 0.05, ns: no significance). Fourthly, as shown in **Figure 8F**, a general upregulation of chemokines (CXCL9, CXCL10, CXCL11) was observed in PTPRD/PTPRT mutant cancers (all *P* < 0.01). In addition, CXCR2 and CXCR4, which are associated with pro-tumor in ICIs, were shown to be lower in PTPRD/PTPRT mutant cancers compared with WT (both *P* < 0.01) (**Figure 8G**). In contrast, CXCR3, an anti-tumor receptor of ICIs, was shown to be upregulated in PTPRD/PTPRT mutant cancers (*P <*0.001) (**Figure 8G**). Furthermore, the pathway enrichment analysis revealed that PTPRD/PTPRT mutations were closely associated with metabolism, including amino-sugar and nucleotide-sugar metabolism, steroid hormone biosynthesis, and fatty-acid metabolism (**Supplementary Figure 3**). In brief, PTPRD/PTPRT mutant patients had higher levels of immune cell infiltration, MHC I expression, and other immune signatures, indicating enhanced anti-tumor immunity in PTPRD/PTPRT mutant patients.

### Construction of a nomogram model to predict the survival benefit of ICIs treatment

**Figure 9:**
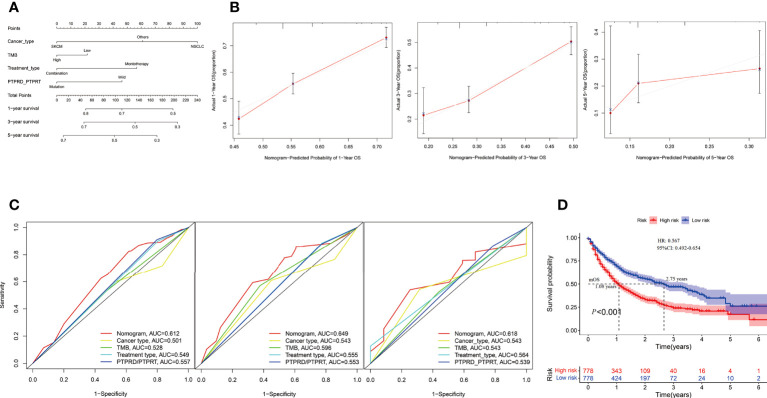
Construction of an integrated prognostic nomogram model. **(A)** Nomogram based on cancer type, TMB, treatment type and PTPRD/PTPRT mutation status of the Samstein cohort. **(B)** Calibration plot of the nomogram for the probability of OS at 1- (left),3- (middle) and 5- (right) years in the Samstein cohort. **(C)** Receiver operating characteristic (ROC) curves for predicting OS at 1- (left),3- (middle) and 5- (right) years in the Samstein cohort. **(D)** Survival curve of OS with the nomogram in the Samstein cohort.

In the Samstein cohort, the univariate analysis showed that cancer type, TMB, treatment type, and PTPRD/PTPRT mutations were statistically significant in predicting OS in patients receiving ICIs. Then, a nomogram model was developed to predict the 1-, 3-, and 5-year survival using above mentioned four parameters in the Smastein cohort **(** **Figure 9A** **)**. The calibration curve confirmed an acceptable level of accuracy **(** **Figure 9B** **)**. The receiver operating characteristic (ROC) curves used for our nomogram, cancer type, TMB, treatment type, and PTPRD/PTPRT mutations are presented in **Figure 9C**. The ROC analysis demonstrated that the nomogram model could accurately distinguish between patients who would benefit from ICIs than cancer type, TMB, treatment type, and PTPRD/PTPRT mutations (area under the curve [AUC] of 1-year survival, 0.612; AUC of 3-year survival, 0.649; AUC of 5-year survival, 0.618). Based on the cutoff value of the risk scores, patients were classified into low-risk and high-risk groups. The survival curves revealed that the low-risk group had a better mOS than the high-risk group (mOS: 2.75 vs 1.08 years, HR = 0.567, 95%CI: 0.492-0.654; *P* < 0.001) **(** **Figure 9D** **)**. Furthermore, the Miao et al. and Huguo et al. cohort was used as an external validation cohort to verify the predictive value of the model. This model proved to have a good level of accuracy (1-year survival, 0.602; AUC of 3-year survival, 0.559; AUC of 5-year survival, 0.610) **(** **Supplementary Figure 4A** **)**, while the low-risk group also showed a better mOS than the high-risk group (mOS: 2.34 vs 1.13 years, HR = 0.650; 95% CI: 0.456– 0.926; *P* = 0.011) in this cohort **(** **Supplementary Figure 4B** **)**.

## Discussion

PTPRD/PTPRT mutations are common mutations in multiple cancer types, but there have only been a few studies that have been conducted on the role of this type of mutations in pan-cancer patients treated with ICIs. In this study, we systematically collected genetic and clinical data to analyze the association between PTPRD/PTPRT gene status and clinical response in pan-cancer patients treated with ICIs. We further validated our findings using another independent ICI-treated cohort and explored the corresponding tumor immune microenvironment (TIME). Moreover, a nomogram model was developed to predict the survival of patients who have received ICI treatment. We found that PTPRD/PTPRT mutations were a favorable biomarker of pan-cancer ICI treatment. Additionally, PTPRD/PTPRT mutations were strongly associated with a higher TMB, MSI score, TCR score, higher levels of immune cell infiltration, and enriched immune-related signatures, indicating an enhanced level of anti-tumor immunity in PTPRD/PTPRT mutant cancer patients. Finally, the nomogram model based on the four factors, cancer type, TMB, treatment type, and PTPRD/PTPRT gene status, indicated a good accuracy in predicting the 1-, 3-, and 5-year survival of pan-cancer patients treated with ICIs.

PTPR, a subfamily of class I PTPs, are involved in protein dephosphorylation, which has been shown to inhibit multiple cellular signaling pathways. Previous studies have demonstrated that PTPRD exerts a suppressive effect in breast cancer and liver cancer (27, 28). Hsu et al. demonstrated that deleterious PTPRT/PTPRD alterations are associated with the shorter progression-free survival of CRC patients who had received bevacizumab (29). Kim et al. suggested that missense mutations in the catalytic domain of PTPRD/PTPRT are implicated in reducing its phosphatase activity (30). Consistently, our study revealed that patients with PTPRD/PTPRT mutations who did not receive ICI treatment in TCGA pan-cancer cohort had a worse clinical outcome, compared with their WT counterparts, indicating that patients with PTPRD/PTPRT mutations may have a worse impact on patients across multiple cancer types.

In this study, prolonged mOS was observed in PTPRD/PTPRT mutant patients who received ICI treatment, compared with the WT, across multiple cancers in two independent ICI-treated cohorts, confirming the prediction ability of PTPRD/PTPRT mutations for the efficiency of ICI treatment. This result is partially consistent with the results of a previous study conducted by Zhang et al., which found that PTPRT mutations may be considered as a potential indicators for assessing ICI efficacy in melanoma, NSCLC, and multiple other cancers (31). Moreover, Zhang et al. demonstrated that better PFS in anti-PD-(L)1monotherapy was associated with PTPRD mutations in non-squamous NSCLC (32). Accordingly, the clinical benefits of PTPRD/PTPRT mutations in patients treated with ICIs may be the result that the clinical benefits of PTPRD/PTPRT mutations in ICI therapy outweigh its harmful impacts on outcomes (**Figure 3**). The increase in TMB has been reported to be associated with a higher neoantigen load, which was associated with a stronger immune response and greater level of immunogenicity (14). Therefore, we further analyzed subgroup survival in patients treated with ICIs based on TMB level and PTPRD/PTPRT gene status. As expected, among TMB-high patients, PTPRD/PTPRT mutant patients had the longest survival, compared with the WT group. These results suggest that among TMB-high patients, PTPRD/PTPRT mutations enhance the response to ICI treatment. Another subgroup analysis conducted based on cancer types showed that PTPRD/PTPRT mutant patients treated with ICIs had significantly longer OS than WT patients in NSCLC and SKCM, which is consistent with the results of previous studies (17, 33). Preclinical studies have suggested that the combination of anti-PD-1 or anti-PD-L1 and antibodies against CTLA-4 is a promising treatment strategy for advanced cancers (34). In this study, ICI monotherapy, including PD-1/PD-L1 inhibitors and anti-CTLA4, showed superior efficacy in PTPRD/PTPRT mutant patients, compared with the WT group. However, comparable mOS was observed between PTPRD/PTPRT mutant and WT patients treated with a combination of anti-PD1/PD-L1 and anti-CTLA4, which may have been due to the small sample size. Checkmate-227 and MYSTIC have proven that high TMB is not effective in predicting the prognosis of patients treated with anti-PD-1 in combination with anti-CTLA4 (35, 36). Therefore, we conjectured that the predictive value of PTPRD/PTPRT differed between monotherapy and combination therapy, and further exploration is warranted.

In this study, PTPRD/PTPRT mutant patients had higher TMB (6), MSI score (8), and TCR score (37), and were positively correlated with ICI treatment. DDR (38) and MMR gene mutations (39), which can increase tumor immunogenicity and promote anti-tumor immunity, were more frequent in the PTPRD/PTPRT mutant cancer patients than in the WT patients. From the above results, it was observed that multiple markers are positively associated with ICI treatment response, which was remarkably higher in the PTPRD/PTPRT mutant cancer patients than in WT patients. It was reported that inactivated PTPRD in gastric cancer can promote angiogenesis by inducing CXCL8 expression (40). In addition, PTPRD regulates PD-L1 signaling in hepatocellular carcinoma (41). The JAK/STAT pathways is important in regulating the differentiation of immune cells and antigen presentation (42), and has been demonstrated to be a critical pathway that is regulated by PTPR (30). Therefore, it is reasonable to speculate that PTPR may influence immunotherapy efficacy by regulating the TIME.

Although previous studies have analyzed the PTPD/PTPRT mutation-associated prognostic value of NSCLC treated with ICIs, as no study has systematically explored the relationship between PTPRD/PTPRT mutations and TIME. In this study, the further enrichment of immune signatures, including CD8^+^ T cells, Tfh, B cells, DCs, cytotoxic activity, and pro-inflammation, were observed in PTPRD/PTPRT mutant cancers, indicated of the formation of an enhanced anti-tumor immune microenvironment (43). In addition, elevated M1 infiltration and decreased M2 infiltration levels were observed in PTPRD/PTPRT mutant cancer patients, which can enhance anti-tumor immunity (44). The increase in immune-stimulators, including CD80 (45), ICOS (46), and various TNFSFs (47), also strengthened anti-tumor immunity. Importantly, higher PD-L1 and CTLA4 expression levels were observed in the PTPRD/PTPRT mutant cancer patients, compared with WT patients, suggesting that ICI treatment is more applicable for PTPRD/PTPRT mutant cancer patients. Other potential immune checkpoints, such as LAG3, CD96, PDCD1, BTLA, IDO1, and TIGIT, were also enriched in PTPRD/PTPRT mutant cancers. As is well known, MHC molecules perform important roles in antigen presentation and TCR recognition, and are critical for anti-tumor immunity (48). Notably, the significant upregulation of MHC I molecules was observed in PTPRD/PTPRT mutant cancers. Furthermore, the expression levels of CXCL9, CXCL10, CXCL11, and their receptor CXCR3, which perform anti-tumor roles in ICI treatment (49), were found to have increased in PTPRD/PTPRT mutant cancer patients. In contrast, the expression levels of tumor-promoting molecules, including CXCR2, CXCR4, CXCR6, and CX3CR1 (49), were found to have decreased in PTPRD/PTPRT mutant cancers. These findings indicate that PTPRD/PTPRT mutations contribute to the development of “hot” tumor with enhanced anti-tumor immunity.

Nomograms and prognostic scores can be used to predict the outcomes of ICI in patients with lung cancer and SKCM (50–52). However, there are currently no models that have been developed to predict the OS in pan-cancer patients treated with ICIs. In this study, we developed and internally validated a nomogram that can be used to predict the survival of pan-cancer patients treated with ICIs. Based on the risk score and nomogram, clinicians can calculate an individual score for each patient and can then predict the 1-, 3-, and 5-years OS of patients treated with ICIs. Our findings may help identify patients who are less likely to benefit from ICI treatment alone and those who are in more urgent need of a novel combination, although this finding should be prospectively validated.

This study demonstrated that PTPRD/PTPRT mutations can be a good predictive biomarker for the efficacy of ICI treatment across multiple cancers. However, several limitations of this study should be noted. First, data on the ICI-treated patients were obtained from public databases, therefore specific information was not available, which may lead to analysis bias. Second, the results may be biased due to different mutation frequencies in different cancers. Third, further analyses need to be conducted in the future due to the small sample sizes of PTPRD/PTPRT mutation patients of certain types of cancers, such as GBM, RCC, and HNSC, included in this study. In addition, the effect of PTPRD/PTPRT mutations on TIME need to be verified through further experiments.

## Conclusion

Overall, the results of this study demonstrated that PTPRD/PTPRT mutations are correlated with a poor prognosis and that PTPRD/PTPRT mutations might act as a pan-cancer biomarker for the prediction of ICI treatment efficacy. Mechanistically, PTPRD/PTPRT mutations are strongly associated with high TMB, high MSI score, and an enhanced anti-tumor microenvironment, thus PTPRD/PTPRT mutations may be a promising biomarker for the prediction of the ICI treatment response in several cancers.

## Data availability statement

The original contributions presented in the study are included in the article/**Supplementary Material**. Further inquiries can be directed to the corresponding author.

## Author contributions

B-C, guidance on study concept and design. XS and WZ, data collection and processing, and manuscript writing. XZ, manuscript language modification. MY, figures revision. JL, statistical analysis. All authors contributed to the article and approved the submitted version.

## Funding

The study was supported by Natural Science Foundation of Shandong Province (ZR202108050037).

## Conflict of interest

The authors declare that the research was conducted in the absence of any commercial or financial relationships that could be construed as a potential conflict of interest.

The reviewer KH declared a shared parent affiliation with the authors to the handling editor at the time of the review.

## Publisher’s note

All claims expressed in this article are solely those of the authors and do not necessarily represent those of their affiliated organizations, or those of the publisher, the editors and the reviewers. Any product that may be evaluated in this article, or claim that may be made by its manufacturer, is not guaranteed or endorsed by the publisher.

## Supplementary material

Supplementary Figure 1 Subgroup survival analysis based on tumor type using ICIs-treated cohort (Samstein et al., n = 1556). Survival analysis in NSCLC **(A)**, SKCM **(B)**, BLCA **(C)**, CRC **(D)**, EAC **(E)**, HNSC **(F)**, GBM **(G)**, RCC **(H)**, respectively. NSCLC, non-small cell lung cancer; SKCM, melanoma; BLCA, Bladder urothelial carcinoma; CRC, colorectal cancer; EAC, esophagogastric cancer; HNSC, head and neck squamous cell carcinoma; GBM, glioblastoma; RCC, renal cell carcinoma.

Supplementary Figure 2 Differences in pathway activities scored by GSEA between PTPRD/PTPRT mutant and WT tumors in TCGA dataset and the most relevant enrichment pathways are shown in the above figure. Blue bars mean that the enrichment score (ES) of the pathway is more than 0. Conversely, yellow bars mean that the ES of the pathway is less than 0.

Supplementary Figure 3 Validation of the nomogram predicting OS in patients treated with ICIs. **(A)** ROC curves for predicting OS of the nomogram in the Miao et al. and Huguo et al. cohort. **(B)** Survival curve of OS with the nomogram according to the risk score in the Miao et al. and Huguo et al. cohort.

## Glossary

**Table:**

BLCA	Bladder urothelial carcinoma
CESC	Cervical squamous cell carcinoma and endocervical adenocarcinoma
CR	Complete response
CRC	Colorectal cancer
CTLA-4	Cytotoxic T lymphocyte antigen 4
DCB	durable clinical benefit
DDR	DNA damage response
DFS	Diseasefree survival
DSS	Disease-specific survival
DUSPs	Dual-specificity phosphatases
EAC	Esophagogastric cancer
ES	Enrichment score
FDA	Food and Drug Administration
GBM	Glioblastoma
HNSC	Head and neck, squamous cell carcinoma
HR	Hazard ratio
ICIs	Immune checkpoint inhibitors
MHC	Major histocompatibility complex
MMR	mismatch repair
MSI	microsatellite instability
NSCLC	Non-small cell lung cancer;
NDB	no durable benefit
OS	Overall survival
PD	Progressive disease
PFS	Progression-free survival
PD-(L)1	Programmed cell death (ligand) 1
PTPN	non-receptor PTPs
PFS	Progression-free survival
PR	Partial response;
PTPs	Protein tyrosine phosphatases
PTPR	Receptor PTPs
PTPRD	Protein tyrosine phosphatase receptor type D
PTPRT	Protein tyrosine, phosphatase receptor type T
PTPRD/PTPRT mutation	PTPRD or PTPRT mutation
RCC	renal cell carcinoma
STAD	Stomach adenocarcinoma
SD	Stable disease
SKCM	Skin Cutaneous Melanoma
ssGSEA	Single-sample gene set enrichment analysis
TCGA	The Cancer Genome Atlas
TCR	T cell receptor
TMB	Tumor mutational burden
WT	wild type
TIME	tumor immune microenvironment.
