# Supplementary figures and images for: PTPRD/PTPRT mutation as a predictive biomarker of immune checkpoint inhibitors across multiple cancer types
Source: Front Immunol. 2022 Sep 29;13:991091. doi: 10.3389/fimmu.2022.991091 (PMC9556668; doi:10.3389/fimmu.2022.991091)

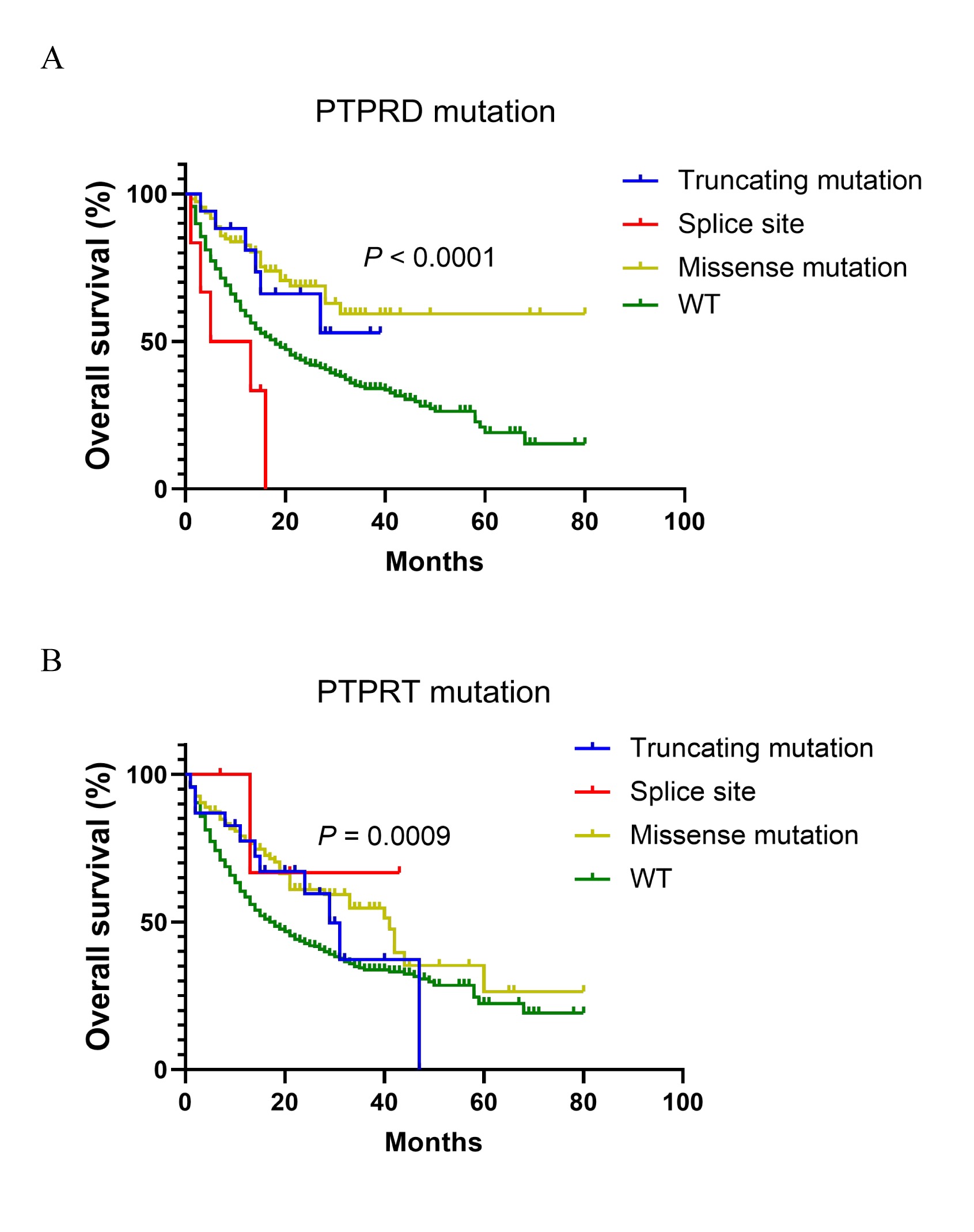

Supplement: Supplementary Figure 1 — Subgroup survival analysis based on tumor type using ICIs-treated cohort (Samstein et al., n = 1556). Survival analysis in NSCLC (A), SKCM (B), BLCA (C), CRC (D), EAC (E), HNSC (F), GBM (G), RCC (H), respectively. NSCLC, non-small cell lung cancer; SKCM, melanoma; BLCA, Bladder urothelial carcinoma; CRC, colorectal cancer; EAC, esophagogastric cancer; HNSC, head and neck squamous cell carcinoma; GBM, glioblastoma; RCC, renal cell carcinoma. [file Image_1.tif]

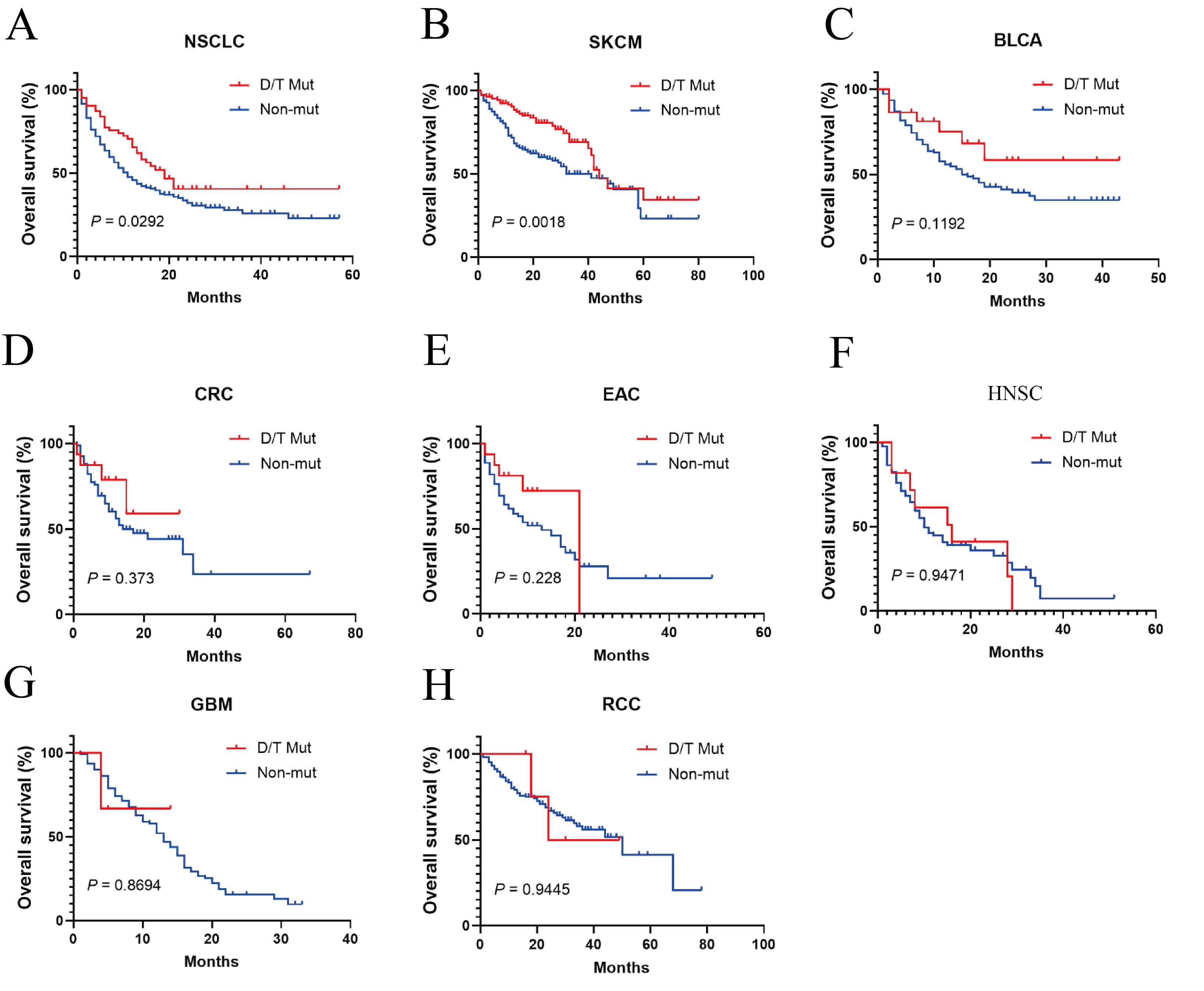

Supplement: Supplementary Figure 2 — Differences in pathway activities scored by GSEA between PTPRD/PTPRT mutant and WT tumors in TCGA dataset and the most relevant enrichment pathways are shown in the above figure. Blue bars mean that the enrichment score (ES) of the pathway is more than 0. Conversely, yellow bars mean that the ES of the pathway is less than 0. [file Image_2.tif]

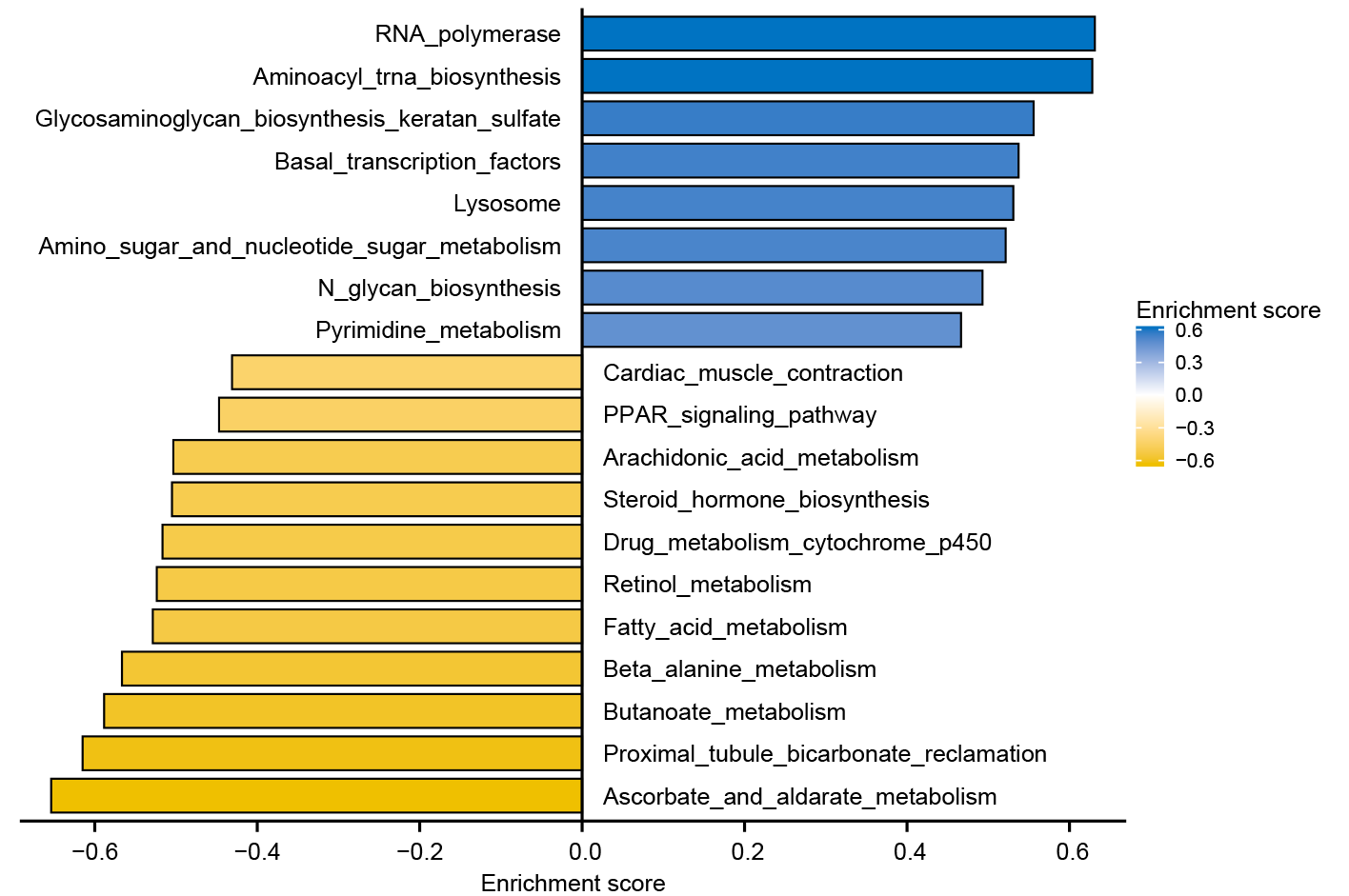

Supplement: Supplementary Figure 3 — Validation of the nomogram predicting OS in patients treated with ICIs. (A) ROC curves for predicting OS of the nomogram in the Miao et al. and Huguo et al. cohort. (B) Survival curve of OS with the nomogram according to the risk score in the Miao et al. and Huguo et al. cohort. [file Image_3.png]

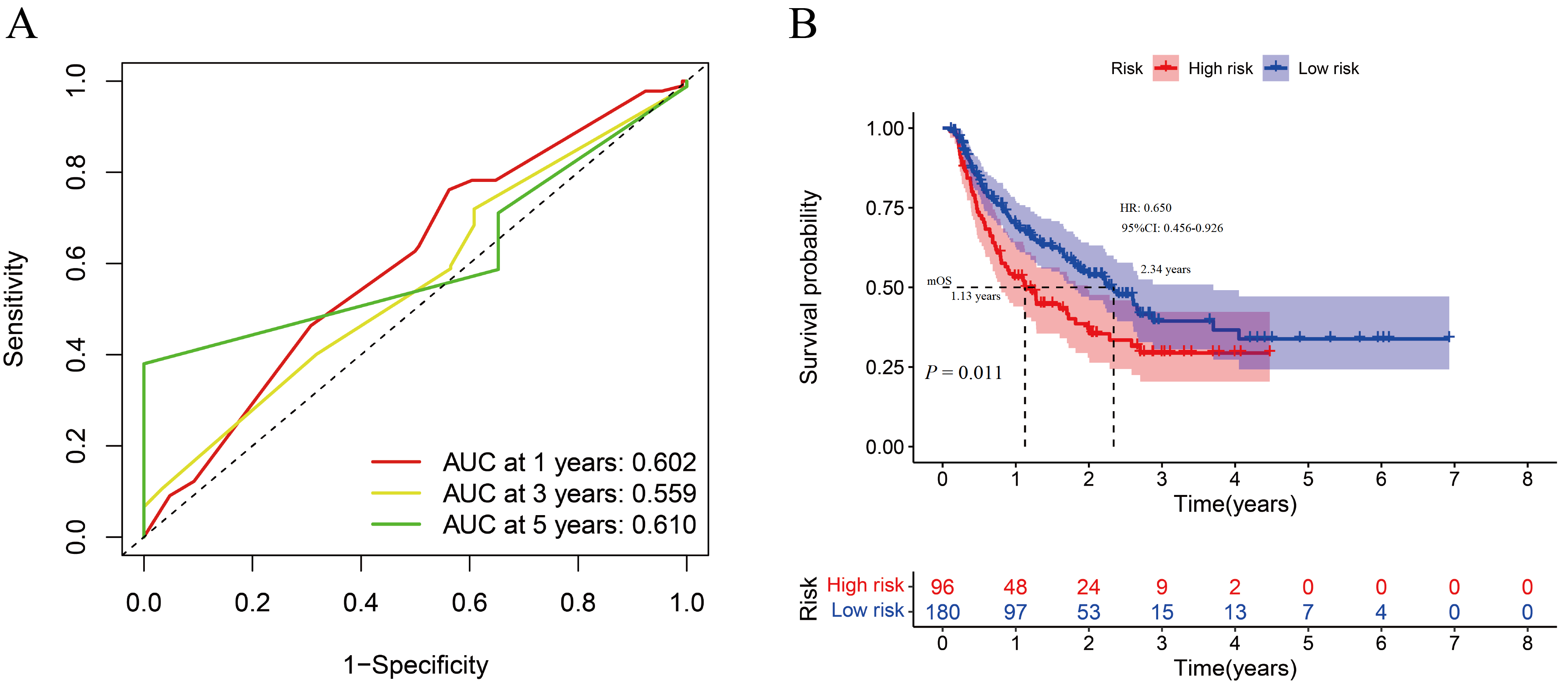

Supplement: Supplementary file 4 [file Image_4.tif]
