# Supplementary material for: PTPRD/PTPRT mutation as a predictive biomarker of immune checkpoint inhibitors across multiple cancer types
Source: Front Immunol. 2022 Sep 29;13:991091. doi: 10.3389/fimmu.2022.991091 (PMC9556668; doi:10.3389/fimmu.2022.991091)
Supplement: Supplementary file 5 [file DataSheet_1.docx]

**Table S1.** **Baseline clinicopathological characteristics in ICIs-treated validation set (Miao et al. and Huguo et al.).**

| **Variables** | **D/T mutation (n=82)** | **Non-D/T mutation(n=195)** | ***P*** |
| --- | --- | --- | --- |
| **Age** |  |  | 0.815 |
| <65 | 32 (49.4) | 75(56.5) |  |
| >= 65 | 26 (50.6) | 56 (43.5) |  |
| Unknown | 24 (50.6) | 64 (43.5) |  |
| **Gender** |  |  | 0.806 |
| Female | 29 (12.9) | 72 (14.6) |  |
| Male | 53 (67.1) | 123(62.4) |  |
| **Cancer type** |  |  | < 0.001 |
| NSCLC | 10 (26.9) | 44 (21.3) |  |
| SKCM | 69 (43.4) | 117 (15.8) |  |
| Others | 3 (29.7) | 34(62.8) |  |
| **Treatment** |  |  | 0.622 |
| Monotherapy | 80 (81.9) | 188 (15.1) |  |
| Combination | 2 (18.1) | 7 (84.9) |  |
| **TMB** |  |  | < 0.001 |
| Low | 12 (6.6) | 127 (56.4) |  |
| High | 70 (90.4) | 68 (43.6) |  |
| **Median OS (months)** | 17.155 | 11.035 |  |

**Table S2. Univariate and multivariate Cox regression analysis in ICIs-treated validation set ((Miao et al. and Huguo et al.).**

|  | **Univariate analyses** | | | **Multivariate analyses** | | |
| --- | --- | --- | --- | --- | --- | --- |
| **Variable** | **HR** | **95% CI** | ***P*** | **HR** | **95% CI** | ***P*** |
| **Age** |  |  |  |  |  |  |
| < 65 | Reference |  |  | Reference |  |  |
| >= 65 | 1.881 | 1.195-2.960 | 0.006 | 1.951 | 1.239-3.072 | 0.004 |
| **Sex** |  |  |  | NI |  |  |
| Female | Reference |  |  |  |  |  |
| Male | 1.236 | 0.864-1.769 | 0.246 |  |  |  |
| **Cancer type** |  |  |  | NI |  |  |
| NSCLC | 0.573 | 0.297-1.106 | 0.097 |  |  |  |
| SKCM | 0.661 | 0.406-1.076 | 0.096 |  |  |  |
| Others | Reference |  |  |  |  |  |
| **Treatment** |  |  |  | NI |  |  |
| Monotherapy | Reference |  |  |  |  |  |
| Combination | 0.593 | 0.147-2.400 | 0.464 |  |  |  |
| **TMB** |  |  |  | NI |  |  |
| Low | Reference |  |  |  |  |  |
| High | 0.749 | 0.536-1.047 | 0.090 |  |  |  |
| **D/T mutation** |  |  |  |  |  |  |
| No | Reference |  |  | Reference |  |  |
| Yes | 0.657 | 0.449-0.961 | 0.030 | 0.612 | 0.417-0.896 | 0.012 |

**Table S3.**

| **Statistical analysis of DDR gene co-mutation with PTPRD/PTPRT (cBioportal, TCGA pan-cancer data, n = 10967)** | | | | |
| --- | --- | --- | --- | --- |
| MMR-gene | PTPRD/PTPRT mut | PTPRD/PTPRT wild-type | *P* value | Q value |
| MLH1 | 8.99%（80/890） | 1.53% (123/8045) | 1.63E-29 | 1.81E-28 |
| MSH2 | 10.39% (77/741) | 1.84% (158/8580) | 7.91E-29 | 8.20E-28 |
| MSH6 | 10.36% (92/888) | 1.88% (157/8372) | 1.50E-32 | 2.11E-31 |
| PMS2 | 11.28% (94/833) | 2.17% (188/8683) | 2.49E-32 | 3.43E-31 |

| **Statistical analysis of MMR gene co-mutation with PTPRD/PTPRT (cBioportal, TCGA pan-cancer data, n = 10967)** | | | | |
| --- | --- | --- | --- | --- |
| DDR-gene | PTPRD/PTPRT mut | PTPRD/PTPRT wild-type | *P* value | Q value |
| ATM | 20.42%（175/857） | 5.34% (501/9387) | 4.64E-46 | 1.80E-44 |
| ATR | 18.06% (164/908) | 4.32% (399/9234) | 2.23E-46 | 8.83E-45 |
| BRCA1 | 12.84% (112/872) | 2.70% (243/9011) | 1.43E-35 | 2.57E-34 |
| BRCA2 | 18.96% (171/902) | 4.54% (418/9209) | 1.75E-48 | 8.01E-47 |
| BARD1 | 7.76% (63/812) | 1.62% (134/8279) | 2.22E-20 | 1.18E-19 |
| BRIP1 | 12.41% (108/870) | 3.19% (286/8972) | 2.75E-28 | 2.71E-27 |
| ERCC2 | 6.76% (57/843) | 2.24% (186/8317) | 1.71E-11 | 4.35E-11 |
| FANCA | 11.04% (92/833) | 2.77% (256/9257) | 4.64E-25 | 3.58E-24 |
| CHEK1 | 5.66% (47/831) | 1.85% (158/8550) | 6.87E-10 | 1.54E-09 |
| CHEK2 | 7.51% (56/746) | 1.91% (166/8678) | 1.82E-15 | 6.42E-15 |
| PALB2 | 9.38% (80/853) | 1.85% (147/7928) | 1.99E-26 | 1.77E-25 |
| MRE11 | 9.39% (77/820) | 1.74% (151/8681) | 2.13E-27 | 1.97E-26 |
| RAD50 | 11.28% (98/869) | 1.85% (164/8842) | 1.01E-37 | 2.09E-36 |
| CDK12 | 14.55% (128/880) | 4.68% (436/9320) | 6.57E-26 | 5.42E-25 |
